# 7. A new diagnosis of systemic lupus erythematosus in an adolescent male

**DOI:** 10.1093/rap/rky031.006

**Published:** 2018-09-20

**Authors:** Emma Luke, Anne Kinderlerer, Katherine Malbon

**Affiliations:** 1 Rheumatology, St Mary's Hospital, London, UNITED KINGDOM; 2 Paediatrics, St Mary's Hospital, London, UNITED KINGDOM

**Introduction:** We describe the primary presentation of systemic lupus erythematosus (SLE) in an adolescent male, with discussion of the challenges of providing appropriate emergency and long term care in this age group, as well as the potential impact of complex social factors on the management of chronic disease. This case emphasises the potential benefits of the extension of dedicated adolescent services in the outpatient setting, such as those seen in Type 1 diabetes and sickle cell disease, into the acute assessment and inpatient settings.

**Case description:** A 16-year-old male patient presented with a one month history of symmetrical polyarthritis primarily affecting the knees, elbows, wrists and hands. His acute presentation was precipitated by a one week history of pleuritic chest pain, fever and malaise. There was no significant past medical history. The patient had been taking paracetamol and ibuprofen regularly with some symptomatic benefit, but there were no regular medications. There was a family history of sickle cell anaemia, but the patient’s haemoglobin electrophoresis was normal. An initial psychosocial assessment, using the HEADDS assessment (Home and Environment, Education and Employment, Activities, Drugs, Sexuality, Suicide/Depression) established that the patient lived alone with his mother who worked as a carer, requiring her to be out of the home during the evening leaving the patient alone. He was an A-level student at a local school with a reliable friendship group. He denied use of alcohol, tobacco or recreational drugs. There was no recent history of foreign travel, the patient was not sexually active and no mood disturbance was apparent. Initial observations demonstrated a low grade fever and tachycardia. Clinical examination revealed bilateral boxing glove hands and bilateral warm, tender and swollen wrists, elbows and knees, with significant intra-articular effusions. There was bilateral tender cervical lymphadenopathy and bronchial breath sounds at the left lung base. There were no cutaneous findings or hepato-splenomegaly. Initial investigations demonstrated raised inflammatory markers associated with microcytic anaemia, thrombocytosis and hypoalbuminaemia. A chest radiograph confirmed left lower lobe consolidation and a small pleural effusion. The renal function was normal, however there was non-visible haematuria and proteinuria on urinalysis and a subsequent protein:creatinine ratio of 47mg/mmol suggested an element of nephropathy. The diagnosis of community acquired pneumonia and inflammatory polyarthritis was clear, but diagnostic debate arose between a primarily infective process with subsequent reactive arthritis and a first presentation of inflammatory disease with increased susceptibility to infection. With potentially contradicting therapeutic strategies, definitive treatment was necessarily delayed to allow further investigation aimed at a unifying diagnosis. Initial treatment with analgesia, non-steroidal anti-inflammatory’s (NSAIDs), intravenous fluids and amoxicillin yielded a degree of clinical stability, but later required escalation to intravenous ceftriaxone and a morphine patient controlled analgesia system. Ultrasound confirmed active synovitis in the knees and extensive tenosynovitis in the hands and wrists. Low complement levels rose the suspicion of SLE and subsequent autoimmune serology confirmed the diagnosis with the following results: ANA positive (1:2560), dsDNA 4800, crithidia luciliae antibody positive and anti-RNP/Sm, anti-60kd Ro and anti-52kd Ro antibodies positive. Rheumatoid factor, anti-CCP antibody and HLA B27 were negative. A comprehensive infection screen ruled out viral infections including HIV, viral hepatitis and enterovirus, and bacterial infection including pneumococcus, mycoplasma and tuberculosis. Once confident that the primary diagnosis was SLE the patient was commenced on corticosteroids (prednisolone 40mg daily) with significant clinical and biochemical improvement over the following days. Maintenance therapy was initiated with weekly subcutaneous methotrexate (MTX) and the patient was discharged with a plan for frequent outpatient monitoring, with the aim of gradual corticosteroid withdrawl. This took place in the specialist adolescent rheumatology outpatient clinic, where patients are seen by both a paediatrician and an adult rheumatologist. We aimed to establish a maintenance regimen through concurrent up titration of MTX, but this was complicated by variable compliance and some difficulty attending outpatient clinic appointments, due to his mother’s work commitments. After premature cessation of corticosteroids and omitted doses of MTX, the patient was re-admitted with an acute flare of lupus polyarthritis, confirmed both clinically and biochemically. He was managed with analgesia, NSAIDs, corticosteroids (20mg Prednisolone daily) and re-establishment of maintenance MTX with addition of hydroxychloroquine (HCQ.) Multiple social factors had demonstrably impacted the patient’s condition and we focused on identification of the important psychosocial factors through an additional HEADDS assessment. This identified low mood and difficulty accepting a new diagnosis of chronic disease. There was little in the way of social support with a single working parent and choosing not to discuss his diagnosis with friends. Psychological support and invitation to an adolescent patient support groups were declined. Discussions suggested a lack of understanding by the patient and family of the significance of SLE; importance of regular specialist review and the implications of poor compliance. We were able to address this through repeated verbal explanations, reinforced with written patient information material. We negotiated practical solutions to overcome compliance issues, such as telephone reminders for medication doses, monitoring blood tests and clinic appointments. The patient was offered telephone support from both the consultant paediatrician and rheumatologist between visits. The GP was contacted directly notifying them of the patient and family’s difficulties, emphasising the importance of long-term treatment and recruiting them to the patients support network, particularly with regards to supplying regular medications and identifying new issues in the community. Within a week of this second discharge, following non-reassuring monitoring blood results, the patient was recalled for admission. Clinical findings were suggestive of on-going significant disease activity with an evolving left sided pleural effusion and left lower lobe pneumonia with respiratory compromise; progressive weight loss and little improvement in SLE serology and cytopenias. The patient was continued on corticosteroids (20mg prednisolone daily), HCQ and MTX and additionally received two weeks of intravenous antibiotics and drainage of the pleural effusion. This admission coincided with the patients A-level examinations, which he remained keen to sit and we actively encouraged. A discharge plan was negotiated between the patient, family, paediatric and rheumatology teams, permitting the patient attendance at exams with daily clinical review and administration of intravenous antibiotics and immunosuppression in the paediatric assessment unit, until two weeks of acute treatment had been completed. This approach proved a successful one and the patient was able to both sit his examinations and complete his acute treatment. Over the last three months, the patient has been established on optimal doses of MTX and HCQ and maintains an excellent record of compliance and attendance for follow up. The corticosteroids have been weaned to 5mg prednisolone daily and the patient is making excellent clinical progress.

**Discussion:** While notable for its clinical interest, this case also emphasises the challenges of providing appropriate emergency and long-term care in the adolescent population; the negative impact of complex social issues on the management of chronic disease and the merits of screening for and addressing these to optimise outcomes. From a clinical perspective, the focus of investigation and management in the early stages of the patient’s presentation was on differentiating between an inflammatory or infective aetiology, allowing safe administration of immunosuppression. Maintenance therapy with corticosteroids, MTX and HCQ was in keeping with British Society for Rheumatology guidelines; however the path to achieving remission was complicated a combination of psychosocial issues. Recognition of these issues prompted sustained psychosocial assessment and support for both the patient and family. Intervention aimed at improving patient and family understanding of SLE and its implications; providing practical solutions to promote compliance; providing a flexible care plan to minimise disruption of schooling and offering psychological support. The progress the patient was able to achieve by tackling these issues is evident, emphasising the advantages of a collaborative, dedicated adolescent service. Selection of the most appropriate care environment and lead clinical team was a particular strength of our patient’s journey. Through identification of his needs, we promptly recognised he had inappropriately been admitted under adult services to an adult ward and arranged for transfer to a paediatric ward. On reflection of similar cases, we have recognised the advantages of a dedicated adolescent service within our trust. With established outpatient adolescent clinics in diabetes, sickle cell disease and epilepsy, many of the resources are already in place for extension into the acute setting. The aim of this service would include rapid identification of ‘best place, best team’ in A&E, collaborative medical and psychosocial assessment and improved transition between paediatric and adult services.

**Key Learning Points**: This case highlights key learning points in both the clinical and psychosocial care of adolescent rheumatology patients. We demonstrate the difficulty in the initial diagnosis of multisystem inflammatory disease, such as SLE, where the differential diagnosis comprises infective versus inflammatory disease, with conflicting therapeutic strategies. The psychosocial aspects of this case in particular have provided a number of important learning points. The application of a thorough psychosocial assessment, such as the H.E.A.D.D.S assessment, allows for identification of important social factors that potentially impact management. Optimisation of these factors can lead to improved patient outcomes. We have aimed to apply these learning points to the improvement of future adolescent services within our trust. A sustained collaborative approach between the paediatric team and adult rheumatology services, both in the inpatient and outpatient setting has produced a good clinical outcome for our patient. We have proposed the potential advantages of widening of the adolescent care services from the outpatient setting to the acute assessment and inpatient setting.

**Disclosure: E. Luke:** None. **A. Kinderlerer:** None. **K. Malbon:** None.

